# The Effect of Chlorhexidine on the Push-Out Bond Strength of Calcium-Enriched Mixture Cement

**Published:** 2014-12-24

**Authors:** Fereshte Sobhnamayan, Alireza Adl, Nooshin Sadat Shojaee, Samina Gavahian

**Affiliations:** a * Department of Endodontics, Dental School, Shiraz University of Medical Sciences, Shiraz, Iran; *; b * Students’ Research Committee, Dental School, International Branch, Shiraz University of Medical Sciences, Shiraz, Iran*

**Keywords:** Bond Strength, Calcium-Enriched Mixture, CEM Cement, Chlorhexidine, Push-out Bond strength, Root-End Filling Materials

## Abstract

**Introduction:** The aim of this *in vitro* study was to evaluate the effect of 2% chlorhexidine (CHX) on the push-out bond strength (BS) of calcium-enriched mixture (CEM) cement. **Methods and Materials:** Root-dentin slices from 60 single-rooted human teeth with the lumen diameter of 1.3 mm were used. The samples were randomly divided into 4 groups (*n*=15), and their lumens were filled with CEM cement mixed with either its specific provided liquid (groups 1 and 3) or 2% CHX (groups 2 and 4). The specimens were incubated at 37^°^C for 3 days (groups 1 and 2) and 21 days (groups 3 and 4). The push-out BS were measured using a universal testing machine. The slices were examined under a light microscope at 40× magnification to determine the nature of bond failure. The data were analyzed using the two-way ANOVA. For subgroup analysis the student t-test was applied. The level of significance was set at 0.05. **Results: **After three days, there was no significant difference between groups 1 and 2 (*P*=0.892). In the 21-day specimens the BS in group 3 (CEM) was significantly greater than group 4 (CEM+CHX) (*P*=0.009). There was no significant difference in BS between 3 and 21-day samples in groups 2 and 4 (CEM+CHX) (*P*=0.44). However, the mean BS after 21 days was significantly greater compared to 3-day samples in groups 1 and 3 (*P*=0.015). The bond failure in all groups was predominantly of cohesive type. **Conclusion: **Mixing of CEM with 2% CHX had an adverse effect the bond strength of this cement.

## Introduction

Calcium-enriched mixture cement (CEM) is a favorable biomaterial for repairing root perforations because of its excellent biocompatibility, sealing ability, hard tissue induction, cementogenesis and PDL formation [1-4]. This cement has antibacterial effect similar to calcium hydroxide [5]. It also has low cytotoxicity similar to mineral trioxide aggregate (MTA) [6]. CEM and tooth-colored ProRoot MTA showed similar sealing ability in repair of furcal perforation [7] or filling the entire canal space prior to root-end resection [8]. CEM cement has an acceptable fungicidal effects against *Candida Albicans* and is able to maintain its effect in concentration of 50 mg/mL after 24 h [9]. Different treatment strategies were applied for sealing perforation with root-end filling materials [10, 11]. 

Depending on circumstances, perforation site can be sealed after or prior to root canal cleaning and shaping [12]. However, since leakage of some irrigants through the perforated area may cause severe irritation of the periodontal tissue during the cleaning and shaping of root canals [13], it has been suggested that the perforation defects should be repaired before complementing endodontic treatment [14]. Following the repair of furcal perforations, and after 7 days of incubation for initial set, endodontic treatment is performed with various irrigation solutions that cause inevitable contact of endodontic irrigants with the site of furcal repair [15].

Chlorhexidine (CHX) is a dicationic bisguanide cholorophenyl ring, that was initially used as a general disinfectant because of its broad antibacterial action [16]. In endodontics, CHX is also used as an irrigant to disinfect the root canal system [17, 18]. It has been shown that mixing MTA with CHX increases the antibacterial efficacy of MTA [19, 20]. A study in rats showed that MTA mixed with CHX caused only a weak inflammatory response on subcutaneous connective tissues, which subsided continuously over time; therefore, the set mixture is considered biocompatible [21]. There are inconsistent results in the literature regarding effect of CHX on the physical properties of MTA. One study reported that MTA mixed with CHX gel did not set even after seven days [22]. In contrast, in the study by Holt *et al.* [19], MTA was mixed with liquid 2% CHX, and after 72 h most samples were set enough to allow performing compressive strength test. Their results revealed that MTA mixed with sterile water had compressive strength higher than that of MTA mixed with CHX. In an *in vitro* study, immersion of dentin disks filled with MTA in CHX had no significant effect on bond strength (BS) of MTA [15].

Different studies have been shown that CEM cement has higher antibacterial properties compared to MTA [5, 23]. Similar to MTA, it has been reported that mixing with CHX increases the antimicrobial effects of CEM cement [24].

The question is whether CHX affects the physical properties of CEM cement, or not. Resistance of dental materials to dislodgment forces is an important factor in the success of different endodontic procedures like repair of perforations, apical barrier formation, and root-end filling. Evaluation of the BS between these materials and dentin will show the value of adhesion between them. Different techniques can be used to evaluate the BS of a dental material to dentin including tensile, shear, and push-out BS tests. In the present study, push-out BS test was used, which is the most reliable method for evaluating the resistance of materials to dislodgement forces based on the results of previous studies [25, 26]. Therefore this study was designed to evaluate the effect of CHX on the push-out BS of CEM cement.

## Materials and Methods

Freshly extracted, single-rooted human teeth, including maxillary incisors and mandibular premolars, were selected and stored in 0.5% chloramine-T before use. All the teeth had mature apices and intact roots. Teeth with cracks or internal resorption were excluded from the study. The crowns of all teeth were removed, and the middle thirds of the roots were sectioned perpendicular to the long axis to obtain 60 dentin disks with a thickness of 1.3±0.2 mm.

The lumens of the dentin disks were prepared with sizes #2 to 5 of Gates Glidden drills (Dentsply Maillefer, Ballaigues, Switzerland), to obtain a standardized diameter of 1.3 mm. To remove the smear layer, disks were immersed in 17% ethylenediaminetetraacetic acid (EDTA), and then in 2.5% sodium hypochlorite (NaOCl), for 3 min each. The samples were then immediately washed in distilled water and dried. The dentin disks were randomly divided into 4 groups (*n*=15), and their lumens were filled with CEM cement (BioniqueDent, Tehran, Iran). In groups 1 and 3 (CEM), CEM cement was mixed according to the manufacturer’s instruction; in groups 2 and 4 (CEM+CHX), CEM was mixed with 2% CHX (Consepsis V, Ultradent Products, Inc., South Jordan, UT, USA).

The specimens were wrapped in pieces of gauze soaked in normal saline and kept in sealed plastic containers. The specimens were the incubated at 37^°^C for 3 (groups 1 and 2) and 21 days (groups 3 and 4).

***Push-out bond strength test***

After the experimental periods, the push-out BSs were measured using universal testing machine (Z050, Zwick GmbH, Ulm, Germany). The dentin disks were placed on a metal slab with a central hole to allow for the free motion of the plunger.

The specimens were loaded with a 0.7-mm diameter cylindrical stainless steel plunger at a speed of 1 mm/min [25]. The maximum load applied to CEM cement before dislodgement, was recorded in Newton (N). To express the BS in MPa, the recorded values in N was divided by the adhesion surface area of CEM cement in mm^2^ calculated according to following formula; 2*πr*×*h*, where *π* is the constant 3.14, *r* is the root canal radius (1.3 mm), and *h* is the thickness of the root slice in mm.

The slices were then examined by the light microscope at 40× magnification to determine the nature of the bond failure. Each sample was categorized into 1 of 3 failure modes; adhesive failure at the CEM and dentin interface, cohesive failure within CEM cement and mixed failure.

Push-out BS data was transformed using natural logarithm to achieve normality. The two-way ANOVA test was used to assess the simultaneous effects of group and time. For subgroup analysis, the student’s t-test was used. The level of significance was set at 0.05.

## Results

Logarithm transformation was done to normalize data. The mean values and standard deviation of push-out BS in four experimental groups are shown in Table 1.

**Figure 1:**
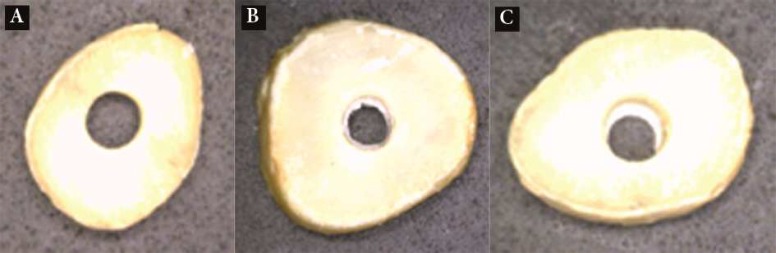
Different failure modes; *A*) adhesive failure at the CEM and dentin interface, *B*) cohesive failure within CEM cement, *C*) mixed failure

There was an interaction effect between all groups (*P*=0.028). Subgroup analysis showed that after 3 days, there was no significant difference between groups 1 (CEM) and 2 (CEM+CHX) (*P*=0.892). However, after 21 days, the BS in group 3 (CEM), was significantly more than group 4 (CEM+CHX) (0.92±0.68 and 0.007±1.06, respectively) (*P*=0.009).

Moreover, there was not a significant difference in BS between groups 2 and 4 (CEM+CHX) (*P*=0.44). Comparison of groups 1 and 3 (CEM) showed that the mean BS after 21 days (0.92±0.68) was significantly greater than that of 3-day samples (0.21±0.81) (*P*=0.015) 

**Table 1:** Mean (SD) of bond strength in different groups

**Group (days)**	**Mean (SD)**	**SD**
**CEM+CHX (3)**	1.55 (0.25)	0.78 (0.73)
**CEM+CHX (21)**	1.63 (0.007)	1.73 (1.05)
**CEM (3)**	1.70 (0.21)	1.52 (0.80)
**CEM (21)**	3.03 (0.92)	1.73 (0.68)

Bond failure in all groups was predominantly of cohesive type, although some samples exhibited mixed and adhesive failure patterns, as well (Table 2) (Figure 1).

## Discussion

This *in vitro* study compared the push-out BS of CEM cement samples mixed with 2% CHX to that of conventionally mixed samples, at two different time intervals and showed that mixing CEM cement with CHX reduces its BS.

The success of furcal perforation repair depends on a well-placed coronal restoration and the resistance of the repair material to displacement forces during condensation of permanent restorative materials. The amalgam condensation force could reach up to 3.7-11.3 MPa during condensation with different pluggers [27]. This pressure is high enough to cause the dislodgement of furcal repair materials [25, 28]. Thus, the BS of the perforation repair materials is an important factor in clinical situations. To assess the BS, various methods have been used including tensile, shear, and push-out BS tests [29]. The push-out bond test has been shown to be practical and reliable [30-33].

The results of the present study showed that there was no statistically significant difference between the push-out BS of groups 1 and 2 (*i.e.* CEM and CEM+CHX) at 3-day interval. This finding is in accordance with those of Yan *et al.* [15] who found that immersion of MTA in CHX for two hours, did not adversely affect MTA-dentin BS. Holt *et al.* [19] also showed that mixing of MTA with 2% CHX had no adverse effect on the compressive strength of MTA after three days.

However, after 21 days the results showed that the BS in group 3 (CEM) was significantly greater than group 4 (CEM+CHX). Increasing the setting time from 3 to 21 days, did not increase the push-out BS of CEM+CHX mixture contrary to that of CEM. 

Evaluation of the effect of time on the BS of MTA in some studies, has shown that in dry conditions there is no significant increase in the BS of MTA but under wet conditions the push-out BS of MTA showed a statistically significant increase from days 3 to 21 [34]. The results of the current study is also partly in agreement with the results of Rahimi *et al.* [31], who reported an increase in the BS of CEM cement mixed with normal saline from 24 h to 7 days although in the current study CEM+CHX group showed an increase in the BS with the pass of time. One reason for the observed discrepancies in groups 1 and 2 in the present study may be related to the probable morphological alteration of the interfacial layer caused by CHX, as it has been reported that CHX caused a significant increase in the amount of the needle-shaped crystals in MTA [15].

Hong *et al.* [35] found that when CHX was used as an irrigant, apparent crystalline structures on the surface of both accelerated and nonaccelerated MTA samples were not observed. The surface crystals had the thin plate structures, and their size was reduced almost to one tenth of those of the control group. In an energy dispersive spectroscopy (EDS) analysis, silicon was detected along with calcium, oxygen, and carbon, which proved that they were not the typical CH crystals. These findings may explain why the push-out BS of the CHX groups was significantly reduced in MTA samples exposed to CHX. This could also be a reason for the lower BS of CHX group in this study; however, scanning electron microscopy (SEM) analysis of CEM cement after exposure to CHX is recommended.

In the present study, the bond failure in all groups was predominantly of cohesive type, although some samples exhibited mixed and adhesive failure patterns (Table 2). This result is in accordance with those of Guneser *et al.* [36] who showed that Dyract AP [a new hydrophilic glass-ionomer cement (DeTrey/Dentsply, Konstanz, Germany)], and Biodentin (Septodont, Saint-Maur-des-Fosses, France) showed predominantly cohesive bond failure of when exposed to different irrigating solutions such as CHX and normal saline. But for MTA the failure pattern was mostly of the adhesive type [36] which is not in accordance with the present study. These differences in the pattern of bond failures might be attributed to different dental materials used in different studies and their different chemical composition and different particle sizes. As CEM cement consists of higher percentage of small particle sizes [37]. Smaller particle size of root-end filling materials like nano MTA showed better physical and chemical properties. It also shows increased surface area and less porosity when exposed to acidic pH levels [38]. The compressive strength of nano MTA is also less affected by acidic environment [39]. The faster setting time of the CEM cement may cause a shorter working time for this material and a faster chemical reaction which is the most important period for structure formation and ion release [40]. 

It has also been shown that CHX can be adsorbed onto hydroxyapatite and teeth (substantivity property of CHX) [31] and it may improve the BS of MTA to dentinal walls [31]. This also could happen in CEM cement samples and cause the bond failure in groups to be of cohesive type. However SEM analysis of CEM cement samples is needed to prove these theories.

**Table 2:** Type of bond failure in different groups

**Group (days)**	**Adhesive (%)**	**Cohesive (%)**	**Mixed (%)**
**CEM+CHX (3)**	13.33	86.66	0
**CEM+CHX (21)**	6.66	93.33	0
**CEM (3)**	6.66	66.66	26.66
**CEM (21)**	0	100	0

## Conclusion

Mixing CEM cement with 2% CHX had an adverse effect on its bond strength. It is therefore not considered a suitable substitute for CEM liquid in clinical situations where the cement may be subjected to dislodgement forces.
